# Carbon isotope evidence for large methane emissions to the Proterozoic atmosphere

**DOI:** 10.1038/s41598-020-75100-x

**Published:** 2020-10-23

**Authors:** Pierre Cadeau, Didier Jézéquel, Christophe Leboulanger, Eric Fouilland, Emilie Le Floc’h, Carine Chaduteau, Vincent Milesi, Julia Guélard, Gérard Sarazin, Amandine Katz, Sophie d’Amore, Cécile Bernard, Magali Ader

**Affiliations:** 1 Université de Paris, Institut de physique du globe de Paris, CNRS, 75005 Paris, France; 2 MARBEC Sète, France, Univ Montpellier, CNRS, Ifremer, IRD, Sète, France; 3 UMR 7245 MCAM, Muséum National D’Histoire Naturelle - CNRS, Paris, France

**Keywords:** Biogeochemistry, Climate sciences, Limnology

## Abstract

The Proterozoic Era records two periods of abundant positive carbon isotope excursions (CIEs), conventionally interpreted as resulting from increased organic carbon burial and leading to Earth’s surface oxygenation. As strong spatial variations in the amplitude and duration of these excursions are uncovered, this interpretation is challenged. Here, by studying the carbon cycle in the Dziani Dzaha Lake, we propose that they could be due to regionally variable methane emissions to the atmosphere. This lake presents carbon isotope signatures deviated by ~  + 12‰ compared to the modern ocean and shares a unique combination of analogies with putative Proterozoic lakes, interior seas or restricted epireic seas. A simple box model of its Carbon cycle demonstrates that its current isotopic signatures are due to high primary productivity, efficiently mineralized by methanogenesis, and to subsequent methane emissions to the atmosphere. By analogy, these results might allow the reinterpretation of some positive CIEs as at least partly due to regionally large methane emissions. This supports the view that methane may have been a major greenhouse gas during the Proterozoic Era, keeping the Earth from major glaciations, especially during periods of positive CIEs, when increased organic carbon burial would have drowned down atmospheric CO_2_.

## Introduction

Both the climate and the oxygenation of the Earth’s surface strongly depend on the carbon biogeochemical cycle. The Earth’s surface oxygenation is believed to have been mainly driven by the burial of organic matter^1^, which is usually estimated by assuming that the carbon isotopic signature of marine carbonates (δ^13^C_carb_) is an indicator of the relative proportions of carbon buried as organic matter or carbonates^2–6^. This conventional approach assumes a carbon cycle at steady state over long timescales with equal C-fluxes entering and exiting the atmosphere–ocean system and C-isotope equilibrium between the atmospheric CO_2_ and dissolved inorganic carbon (DIC) in the ocean. Based on this broadly accepted postulate, δ^13^C_carb_ is assumed to be spatially homogeneous and its evolution over geological times was historically mainly interpreted as reflecting global carbon cycle changes linked to organic carbon burial rate fluctuations^4–6^. However, as more data become available significant spatial variations in the amplitude and the timing of many of the positive carbon isotopic excursions (CIEs) are revealed^7–15^, especially during Proterozoic times, which imply that they are at least in part regionally controlled^14–16^. Improving our knowledge of the mechanisms behind these regional controls could not only change our understanding of some positive CIEs but also our vision of the carbon cycle evolution over geologic times and of its links with major biogeochemical changes in the early Earth’s surface.

The Lomagundi Jatuli Event (LJE, ~ 2.3 to ~ 2.1 Ga^7^) is one of the best examples of this reasoning. It corresponds to a time period recording the most extreme positive CIEs in Earth’s history in terms of duration and amplitude, and is widely assumed to reflect a global event driven by an increase of organic carbon burial^4–10^. Yet, this interpretation remains to be confirmed by independent geological evidence. It implies a huge increase in organic carbon burial rate^5^, recently proposed to be unrealistic^6^_,_ although theoretically possible, and for which direct evidence is still lacking^17^. It also implies an important oxygenation increase, and while recent works seem to indicate that oxygen concentration was indeed appreciably higher during the Paleoproterozoic^18,19^, the temporal coincidence between oxygenation and the LJE is not clearly established yet^20–22^. Moreover, significant spatial variations in the CIEs of the LJE were highlighted, not only in amplitude but also in timing and duration^7^. Several authors have thus proposed that these CIEs could in fact be local or regional occurrences^11–13^, possibly resulting from processes involving methanogenesis activity^11,12^, challenging the idea that they record a global C-cycle perturbation. Others now consider that the LJE remains a global event, while acknowledging that regional controls, remaining to be identified, may modulate it^7,10^.

Our objective here is to investigate the idea that extensive methanogenesis in the water column of restricted environments, when associated to methane escape to the atmosphere^14,15^, may be responsible for pushing δ^13^C_carb_ towards more positive values at a regional scale. We focus on this mechanism not only because, as previously suggested, it might be particularly relevant for understanding the spatial and temporal variability of Proterozoic CIEs, but also because it is one of the potential sources of greenhouse gas to the atmosphere of the early Earth. If the temperatures of the Proterozoic oceans were similar^23^ or higher than on actual Earth^24,25^, then the lower radiation levels from the young sun must have been counterbalanced by a greater proportion of greenhouse gases in the atmosphere^26,27^, possibly including methane, although direct geological evidence for them is still lacking.

In this study, we examine these questions through the lens of a unique thalasso-haline lake that exhibits extremely positive δ^13^C values both in its organic and inorganic carbon pools, and analogous in many ways to some Proterozoic restricted settings.

### A unique thalasso-haline volcanic crater lake

Dziani Dzaha is a shallow tropical volcanic crater lake on the Petite Terre Island of Mayotte (Comoros Archipelago, Indian Ocean)^28^, which is located 50 km west of a huge submarine volcano triggered in 2018^29^. Several surveys have been conducted to determine the lake biological, physical and chemical characteristics^30–34^ (see details in Supplementary Information and Supplementary Fig. S1). The water column is seasonally stratified at about 2 m depths due to precipitation/evaporation-induced salinity changes in surface waters. During stratified periods, salinity and alkalinity increase below the chemocline (from 35psu and ≈ 0.1 M above it to 70 psu and ≈ 0.2 M below), pH decrease from 9.5 to 9, DIC content decrease from ≈ 0.2 to ≈ 0.1 M, dissolved oxygen and sulphate (≈ 3 mM) are totally consumed, reduced species accumulate (e.g. HS^−/^H_2_S up to ≈ 6 mM), and the photosynthetic microorganisms present in the surface waters are replaced by a dense and diverse population of archaea and heterotrophic bacteria at depth. During non-stratified periods, most physical, chemical and biological parameters are constant with depth, except for dissolved oxygen that is only present down to about 1.5 m depending of the photosynthetic activity. The water temperature is of ≈ 30 °C with little depth and seasonal variations. The ecosystem biomass is extremely high^30^ and it is limited to microbial organisms^34^, with stromatolitic constructions thriving in the shallow parts of the lake (Supplementary Information).

Several of the Dziani Dzaha Lake characteristics can be seen as analogous to specific Proterozoic times and environments. The Precambrian oceans salinity was commonly estimated between once and twice that of the modern oceans, with values decreasing with time, assuming that evaporites and brines accumulated progressively on the growing continents^24^. More recently, however, Archean ocean salinity was proposed to be in fact similar to the modern ocean based on the composition of Archean fluids inclusions, challenging the hypothesis of an ocean salinity decrease with times^35^. In any case, based on Phanerozoic ocean salinity reconstructions, which indicate salinity fluctuations from 35 to 50psu^36^, a similar range of variations can be expected throughout Proterozoic times, in good agreement with the lower range of the Dziani Dzaha salinity (i.e. 35 to 70 psu). The recent ocean paleotemperatures reconstructions over Earth’s history are also quite variable. The latest studies based on oxygen isotopic compositions of Precambrian cherts propose a global decrease from 50–60 °C in the Archean to 0–15 °C for the late Phanerozoic^25,37,38^, with a 30–50 °C range in the early Proterozoic (i.e. 2.1–2.3 Ga) and 15–30 °C range in the late Proterozoic (i.e. 0.5–1.5 Ga) which is consistent with the Dziani Dzaha temperature of ≈ 30 °C. A recent study based on oxygen isotopic compositions of marine iron oxide suggest a more temperate and stable climate over the past 3.5 Ga^23^, in which case the Dziani Dzaha temperature would be analogous to that of equatorial environments only. According to recent modelling studies, the evolution of paleo-pH through geological times increased progressively from slightly acidic to slightly alkaline conditions, with a neutral value around 7 at the Archean-Proterozoic boundary increasing to 7.9 at the Proterozoic-Phanerozoic boundary^39–41^. The Dziani Dzaha pH value is thus higher than currently accepted for Proterozoic open oceans but could be analogous to some restricted or lacustrine alkaline Proterozoic environments^42^. Similarly, Precambrian DIC concentrations are suspected to have decreased through Precambrian times^43^, and recent modelling studies proposed values comprised between 25 and 75 mM^44^, lower than the reported DIC concentration in the Dziani Dzaha (i.e. 0.1–0.2 M). Both Archean and Proterozoic oceans are assumed to present sulphate concentration under the mM range^45^, likely below 400 μM and possibly as low as 100 μM throughout most of the Proterozoic. Two specific Proterozoic intervals, however, are suspected more sulfate-rich than the rest of the Proterozoic with sulfate concentrations potentially reaching 1 to 10 mM, i.e. around the Lomagundi-Jatuli Event and during the late Neoproterozoic^45^, which could be in good agreement with sulphate concentration in the Dziani Dzaha surface waters (≈ 3 mM). As to the water column redox structure, in the Dziani Dzaha dissolved O_2_ is limited to the first meter of the water column, while the deeper part of the water column is permanently anoxic and seasonally euxinic when the lake is stratified. Although the Precambrian ocean is widely assumed to have been mostly ferruginous, it was suggested that high productive continental shelves experienced euxinic conditions^19,46–49^. Hence, the Dziani Dzaha Lake redox structure may be another good analogy for Proterozoic environments to which we compare it, i.e. lacustrine, interior seas or restricted epireic seas. Finally, the microbial ecosystem of the Dziani Dzaha dominated by prokaryotes is also a good analogue for most of the Proterozoic time^50,51^, the earliest reliable evidence of eukaryotes are observed in rocks records at 1.7 Ga^50^: the earliest widely accepted photosynthetic eukaryotes evidence are dated at 1.1 Ga^50^, while Metazoan are thought to have evolved only in the late Proterozoic, by 800–750 Ma, together with a major^-^ increase in eukaryotes diversity^51–53^. Stromatolites thrive in the shallow waters of the lake^32^, as they did in Precambrian oceans^54^.

Although some of the Dziani Dzaha Lake features preclude any comparison with Precambrian open ocean (e.g. size and bathymetry, strong primary production, euxinia), many other features support the use this lake as a new modern analogue to putative Proterozoic lakes, interior seas or restricted epireic seas (e.g. temperature, salinity, sulphate scarcity, water column redox, microbial ecosystem). Most importantly, the Dziani Dzaha also presents very positive δ^13^C_carb_ values (see results below) that match the highest CIEs of the LJE and of those following the Neoproterozoic glaciations during early and late Proterozoic times, respectively.

### Constraining the carbon cycle in this unique ecosystem

**Figure 1:**
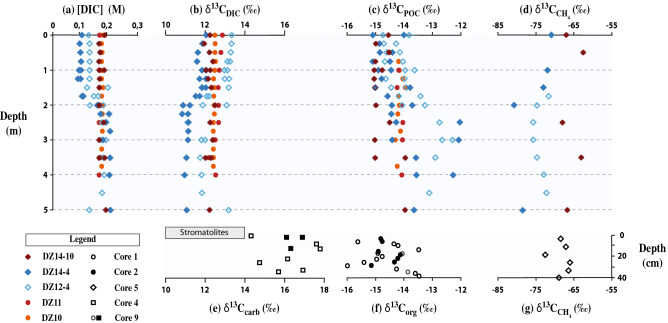
Compilation of carbon concentration and isotopic composition in the Dziani Dzaha. In the water column, (**a**) concentration and (**b**) isotopic composition of dissolved inorganic carbon ([DIC] and δ^13^C_DIC_), (**c**) isotopic compositions of particulate organic carbon (δ^13^C_POC_), (**d**) isotopic composition of dissolved methane (δ^13^C_CH4_). In the sediment columns, (**e**) isotopic compositions of stromatolites and sedimentary carbonates (δ^13^C_carb_), (**f**) sedimentary organic matter (δ^13^C_org_), and (**g**) methane dissolved in the porewaters (δ^13^C_CH4_).

We determined the carbon isotope composition of most of the lake C-pools (Fig. 1 and Supplementary Tables S1 to S4 and S6). In the water column, the dissolved inorganic carbon isotopic signature (δ^13^C_DIC_) was very positive and almost constant at + 12.1 ± 0.6‰. It was constant throughout depth when the water column was non-stratified, and slightly more positive above the halocline (~ + 13‰) and slightly less positive below the halocline (~ + 11‰) during stratified periods (Fig. 1). The suspended particulate organic carbon isotopic signature (δ^13^C_POC_) was constant with depth for both periods at − 14.1 ± 0.8‰. The difference between the isotopic signatures of δ^13^C_DIC_ and δ^13^C_POC_ (~ 26‰) is consistent with the isotope fractionation during C3 photosynthesis. In four sediment cores collected at various locations in the lake, the averaged carbonate carbon isotopic signature (δ^13^C_carb_) was + 16.2 ± 1.1‰ and the averaged organic carbon isotopic signature (δ^13^C_org_) was − 14.5 ± 0.7‰, in good agreement with the contemporaneous δ^13^C_DIC_ and δ^13^C_POC_. The stromatolite δ^13^C_carb_ ranged from + 10 to + 14‰, also consistent with the contemporaneous δ^13^C_DIC_. The methane carbon isotopic signature (δ^13^C_CH4_) was very negative with an average value of − 68 ± 3‰ and − 71 ± 6‰ for dissolved methane within the pore water sediment and the water column, respectively (Fig. 1, Table S2 and S3), and − 65‰ for methane degassing from the lake to the atmosphere (Table S4). While a significant proportion of the produced methane is most likely oxidized in the Dziani Dzaha surface waters, the carbonates observed in surface sediments do not show any isotopic evidence of precipitation from oxidized methane, i.e. more negative value, nor does the dissolved inorganic carbon in the pore water sediment. This suggests that methane oxidation occurs in the water column rather than surface sediment and that, because of the high DIC concentration in the waters of the Dziani Dzaha, the contribution of the low δ^13^C_DIC_ from methane oxidation is strongly diluted.

Overall, our isotope results show not only that the present-day carbon cycle in the lake is accurately recorded in its sediment isotope record, but also that it has been at steady state for more than three hundred years (see Supplementary Information). So far, none of the few known present-day lacustrine systems with ^13^C enrichment exhibit consistent carbon isotope signatures between the lake waters and the sediment, nor convincing evidence for the existence of a steady state^55–60^. In addition, the carbon cycle in these lakes has not been determined well enough to allow an unambiguous evaluation of the processes responsible for the reported ^13^C enrichment. This leaves the Dziani Dzaha as the best analogue identified so far for such an evaluation because it is the only one for which it is legitimate to use a simple steady-state box model to describe its carbon cycle and identify the biogeochemical processes involved in its ^13^C enrichment. The mass and isotope balance of input and output carbon fluxes to and from the DIC in the lake follows the Eq. (1) (see full development in Supplementary Information).

1 $$f_{{{\text{input}}}} \times\updelta ^{{{13}}} {\text{C}}_{{{\text{input}}}} {-}f_{{{\text{output}}}} \times\updelta ^{{{13}}} {\text{C}}_{{{\text{output}}}} = \, 0$$

**Figure 2:**
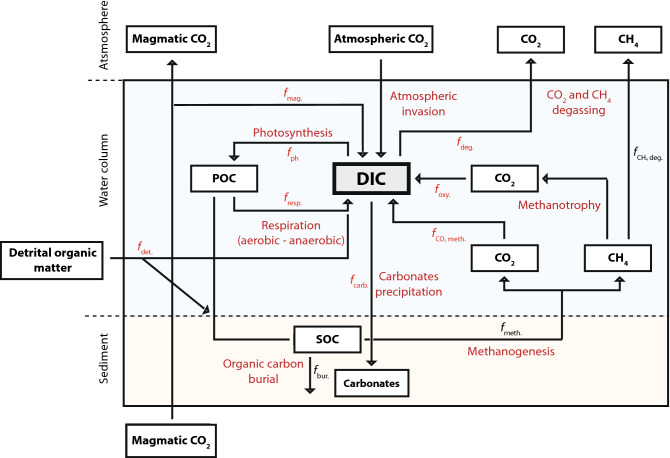
Schematic representation of the main carbon cycling pathways in the Dziani Dzaha. The water column is coloured in blue and the sediment in light brown. Each box represents a carbon pool and arrows represent the different processes that exchange carbon in the lake (details in Supplementary Information).

Carbon fluxes and isotopic signatures for each carbon pool are expressed in mmolC m^−2^ d^−1^ and as ‰ respectively, and are summarized in Table 1 and Fig. 2 (see "Methods" for details of the model and fluxes constraints). We performed direct quantifications of the rate of organic carbon burial (i.e. average value of 5.5 mmolC m^−2^ d^−1^), Growth primary productivity and oxic respiration rate (i.e. average value of 404 mmolC m^−2^ d^−1^ and 214 mmolC m^−2^ d^−1^, respectively) and CO_2_ and CH_4_ net diffusive emission to the atmosphere (i.e. average value of 123 mmolC m^−2^ d^−1^ and 77 mmolC m^−2^ d^−1^, respectively). Interestingly, although the ebullitive contribution in methane degassing is dominant in most modern anoxic lacustrine system^61,62^, in the Dziani Dzaha the average value of its contribution is about 18 ± 15% and the diffusive contribution is predominant (see Supplementary Figure S5). The remaining fluxes were calculated by isotopic mass balance. Some δ^13^C signatures are also expressed as the sum of δ^13^C_DIC_ and an enrichment factor, which is either taken from the literature or determined from direct measurements. The only assumptions considered in this model are i) a methanogenesis degradation pathway with equal CO_2_ and CH_4_ production rates, and ii) 64% of the methane produced by methanogenesis being lost to the atmosphere. This latter amount corresponds to the upper limit used in the literature^55^, and was chosen because shallow waters and anoxia favour methane loss to the atmosphere by reducing its residence time in the water column and the hence the extent of methanotrophy.

**Table 1:** Fluxes and isotopic compositions of inputs and outputs of carbon in Dziani Dzaha.

Inputs				Outputs			
Parameter	Notation	Flux (mmolC m^2^ d^1^)	δ^13^C or ε* (‰)	Parameter	Notation	Flux (mmolC m^2^ d^1^)	δ^13^C or ε (‰)
Respiration	*f*resp	214^a^	0^a^*	Photosynthesis	*f*ph	404^a^	26.2^a^*
CO_2_ methanogenic	*f*_CO2-meth_	120^b^	53.6^b^*	CO_2_ degassing	*f*deg	123^a^	6.2^a^*
CH_4_ oxidation	*f*oxy	43^b^	4.7^a^*	Carbonate precipitation	*f*carb	1.9^a^	4.1^a^*
Detrital organic matter	*f*det	133^b^	− 26.7^a^				
Magmatic CO_2_	*f*mag	18^b^	− 2.7^a^				
Flux balance			528	528			
Calculated δ^13^C_DIC_			12.1‰				

*Indicates the ε values.

^a^Fluxes and isotopic signatures measured.

^b^Fluxes and isotopic signatures constrained by isotopic mass balance calculation.

**Figure 3:**
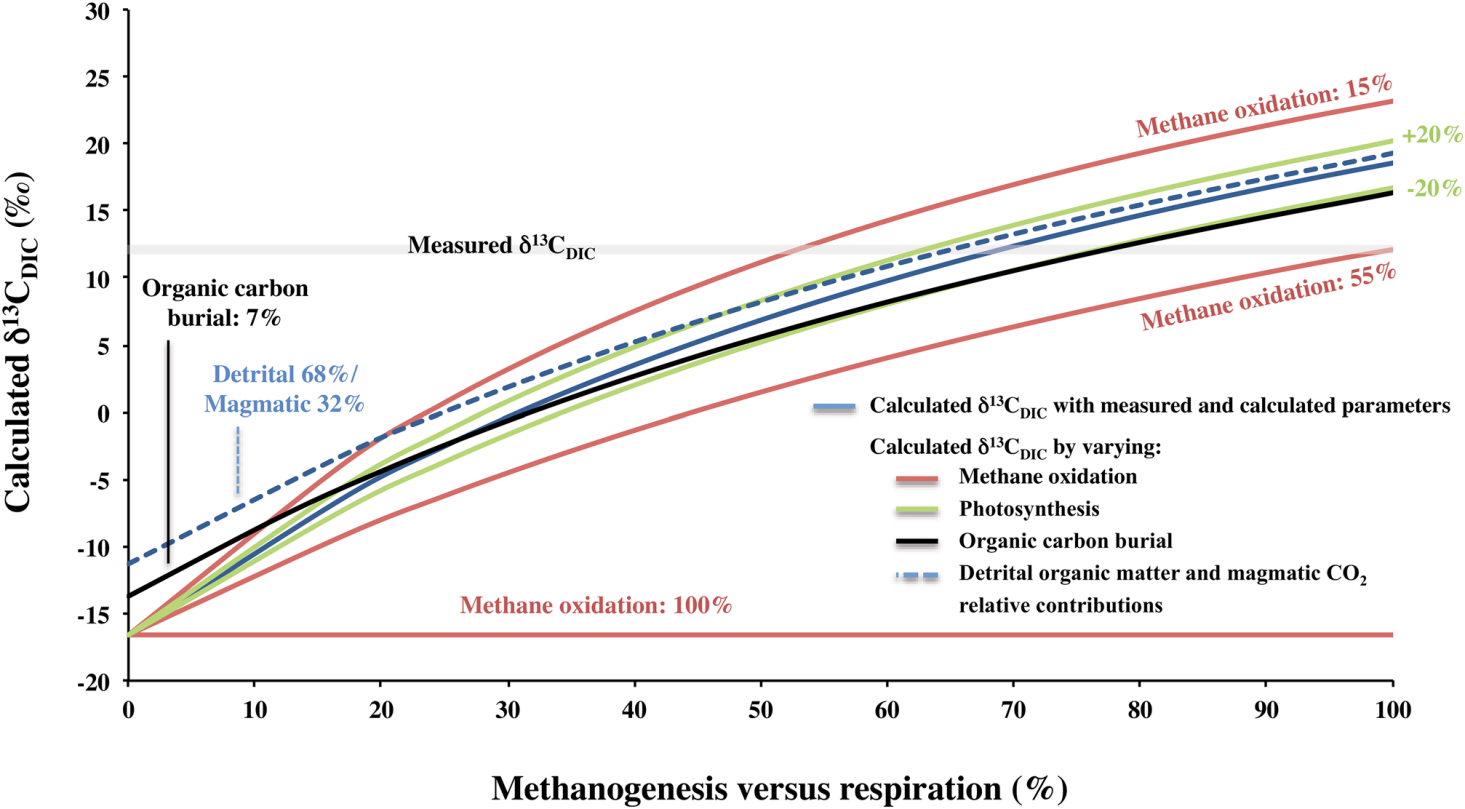
Modelled δ^13^C_DIC_ in the lake water as a function of the relative contribution of respiration and methanogenesis in the remineralisation of the gross primary production. The solid blue line represents the δ^13^C_DIC_ modelled considering the measured and calculated parameters reported in the Table 1 (see "Methods" section), and a proportion of methane oxidized in the water column of 36%. The other curves are representative examples of sensitivity tests to illustrate the influence of each process on the modelled δ^13^C_DIC_. The red curves show various proportions of methane oxidized in the water column. The green curves show photosynthesis fluxes decreased or increased by 20% compared the solid blue line. The black curve shows a percentage of organic carbon burial relative to gross primary production of 7%, to be compared to the 1.4% of the solid blue line. The blue dotted curve shows an alternative assumption with a relative contribution of detrital and magmatic inputs of 68% and 32% respectively (details in Supplementary Information). The grey band corresponds to the average measured isotopic signatures of DIC in the lake.

Sensitivity analyses were performed to evaluate the influence of each process on δ^13^C_DIC_, including CO_2_ and CH_4_ production rates and the proportion of methane lost to the atmosphere (Supplementary Information). The range of possibilities shown by the sensitivity tests unambiguously demonstrate that the fraction of methane lost to the atmosphere is the most important parameter controlling δ^13^C_DIC_. None of the other processes usually proposed to explain high δ^13^C_DIC_ values, such as CO_2_ degassing, oxygenic photosynthesis or carbon burial^56,58–60^, can account for the measured δ^13^C_DIC_ (Fig. 3, and Supplementary Figures S2 to S4). To our knowledge, methane emissions rates from the Dziani Dzaha Lake are among the largest reported for lakes^63^. This probably results from the very thick oxic layer (i.e. 2 m), which limits methane exposure to oxidation, from the high primary productivity largely degraded by methanogenesis, but may be also from methane produced by cyanobacteria within the oxic layer itself. The ability of cyanobacterial communities to produce methane even in oxic environments is increasingly documented and has been shown to increase together with primary productivity^64^. Cyanobacteria are the major primary producers in the Dziani Dzaha Lake; it is thus possible that they contribute to methane production and emissions.

### Implications for the proterozoic carbon cycle

The present study supports the idea that positive CIEs, like those of the LJE, may reflect regionally active methanogenesis with methane emissions to the atmosphere, as previously proposed^14,15^. This hypothesis remains fully compatible with the consensual view that at least part of the amplitude of positive CIEs results from a global increase of organic carbon burial by accounting for at least part and possibly most of the variability in the positive CIEs of the Proterozoic sedimentary record^4–6^. The Dziani Dzaha is a small lacustrine system, but an interior sea (e.g. the Black sea or Caspian sea) with a similar functioning would produce up to 10% of the annual methane flux to the atmosphere suggested to regulate the climate^45^. This implies that methane emissions from restricted basins at the time of CIEs may have compensated the drawdown of atmospheric CO_2_ (should increased organic carbon burial have occurred) keeping the Earth from major glaciations.

This hypothesis nonetheless raises the question of why CIEs induced by regionally strong methane emissions would be abundant only during the early Proterozoic and the Neoproterozoic. Sulphate concentration is assumed to have remained very low during the whole Precambrian, so that low sulphate content cannot be the only controlling factor. In fact, methane emissions can impact δ^13^C_DIC_ value only if its emission rates are high enough to dynamically maintain isotope disequilibria between atmospheric CO_2_ and water DIC. In turn this requires a high methane production and hence a high primary productivity. Strong primary productivity might be more easily reached in restricted environments, e.g. under volcanic influences that can supply ample nutrients, as in the Dziani Dzaha Lake, or within productive continental shelves suspected to have experienced periodic euxinic conditions. In addition, these two specific Proterozoic periods were assumed to be more oxygenated, which might also favour nutrients supply through increased oxidative weathering rates. The paleogeography may thus one of the key factors in the establishment of restricted environment, e.g. interior seas or restricted epireic seas, could thus have a significant influence on the occurrence of CIEs, as suggested for example in the case of the Neoproterozoic positive δ^13^C excursions of the Bambui Group, Brazil^65^.

Beyond their importance in explaining the CIEs variability, our results also have implications for the understanding of the carbon cycle and the climate regulation in the whole Proterozoic. For example, extrapolating the results obtained on the Dziani Dzaha allow us to set an upper limit for the organic carbon burial rate. In Dziani Dzaha, the fraction of the net primary production that is buried and preserved in the sediments is of 2.9%. This is only higher than the maximum value estimated in the Black Sea, which is another euxinic system overlain with at least 60 m of oxic water, where organic matter burial was estimated to 0.5–1.8%^61,66^ (0.1% in the present-day oceans). In Dziani Dzaha, the very high productivity, the very thin oxic surface layer, the low sulphate concentrations, the shallow water column and the high sedimentation rate (close to 1.2 mm year^−1^)^30–34^, all create highly favourable conditions for the preservation and burial of organic matter^67–69^. By analogy, the value of 2.9% could be thus considered as a maximum for the organic burial rate in Precambrian oceans that were also microbially dominated, anoxic and sulphate-poor. This means that most of the net primary productivity was mineralized and, by analogy with the Dziani Dzaha, largely by methanogenesis, even if rates of methanogenesis and methane emissions (and therefore of primary productivity) were not high enough to significantly impact the δ^13^C_DIC_ value in most depositional environments. In this sense, the deciphering of Dziani Dzaha biogeochemistry provides a strong support to the idea recently reassessed^45^ that because of the very low sulphate and oxygen concentrations envisioned in Proterozoic seawater, the Proterozoic oceans could have been a large source of methane to the atmosphere (60 to 140 Tmol y), contributing to the greenhouse climate regulation as the time. It also allows to refute the opposite conclusions previously reached based on an analogy with the ferruginous and sulphate-poor Matano Lake, in which methanogenesis activity was found to be relatively inefficient in mineralizing organic matter^70,71^.

In summary, our geochemical study of the Dziani Dzaha demonstrates that microbial methanogenesis can be responsible for a combination of strong positive carbon isotopic deviation in organic and inorganic pools, and strong methane emissions to the atmosphere. These results illustrate how impactful methanogenesis activity can be at a local scale in modern systems, while many features of Dziani Dzaha allow us, by analogy, to extrapolate its functioning at least to some restricted environments in the Proterozoic. We thus propose that the efficient remineralisation of organic matter by methanogenesis coupled to methane emissions to the atmosphere may be key features of the carbon cycle during Paleo- and Neo-Proterozoic CIEs (some of them being possible proxies for especially high methane fluxes in lakes, interior seas or restricted epireic seas), and possibly of the entire Proterozoic, supporting the hypothesis that biogenic methane may have been a main contributor of the climate regulation at the time.

## Methods

### Field sampling for C isotope analyses

The isotope signatures in this study are based on samples from surveys performed in September 2010, September 2011, April 2012, April 2014 and October 2014. A consistent nomenclature was used to identify each sample taken during the various surveys. For example, for the sample ‘DZ14-4 CLB 2 m’, ‘DZ’ indicates Dziani, ‘14–4′ indicates the year (2014) and the month (April) of the survey, ‘CLB’ refers to the water column station as shown on Supplementary Figure S1 and ‘2 m’ is the depth. Sediment cores were serially numbered from the first survey.

Water samples were collected using horizontal 1.2L Niskin bottles along a vertical profile at the CLB station for each survey (Supplementary Figure S1 and Supplementary Table S1). For each sampling depth, duplicate 12 mL EXETAINER LABCO glass tubes were filled directly from the Niskin bottle and fixed with 0.2 mL of saturated HgCl_2_ solution for δ^13^C_CH4_ analysis. Subsamples were also taken in 500 mL plastic bottles. These subsamples were then filtered under pressure onto pre-combusted Whatman GF/F glass fibre filters (0.7 µm) for δ^13^C_POC_ analysis and several 12 mL EXETAINER LABCO glass tubes were filled with the filtrate and fixed with 0.2 mL of saturated HgCl_2_ solution for DIC and δ^13^C_DIC_ analyses.

During the field trips in April 2012 (DZ12-4) and October 2014 (DZ14-10), several sediment cores were taken. A Uwitec gravity corer (90 mm diam. PVC tubes) was used to collect sediment cores, except for C1 and C2, which were taken by scuba divers using hand tubes (April 2012). Cores C1, C2, C4 and C9 were used for quantifying organic matter and carbonates and for carbon isotope analyses (Supplementary Table S1). They were cut in 3 to 5 cm thick segments and stored in plastic bags, which were themselves enclosed in an air-tight sealed bag (133 µm PROTECTIVE PACKAGING) containing ANAEROCULT patches to maintain anoxic conditions during transport and cold room storage before laboratory analysis. The core C5 was dedicated to dissolved methane carbon isotopic signature analysis in the pore water. Pore water samples were collected immediately after the core retrieval, on the lake shore, using RHIZON sampler at 5 cm intervals immediately after core retrieval.

The gases bubbling up at various locations in the lake were sampled for δ^13^C_CH4_ and δ^13^C_CO2_ analyses (Supplementary Table S1). The samples were taken manually at about 10 cm depth in the water column using 12 mL EXETAINER LABCO glass tubes pre-filled with lake water. They were left open underwater above the source of bubbles until the gas bubbles progressively replaced the water and then closed. The samples were then fixed with an injection of 0.2 mL of saturated HgCl_2_ solution into the remaining water. Gases diffusing through the water–air interface were collected using a purpose-built accumulation chamber^72,73^. The gases were sampled with a syringe through a septum on top of the chamber after 2–4 days of deployment and then injected into evacuated EXETAINER LABCO glass tubes for storage.

### Isotopic analyses

#### DIC and δ^13^C_DIC_ analyses of the water

DIC concentrations and δ^13^C_DIC_ were measured using an AP2003 mass spectrometer on 89 water samples (Supplementary Table S2). First, phosphoric acid (100% H_3_PO_4_) was injected into 12 mL EXETAINER LABCO tubes that were then flushed with helium. For each sample, 0.1 mL water was sub-sampled using two needles and helium to limit contact with air and transferred into the acidified EXETAINER LABCO tubes. The samples were agitated for 15 to 24 h so that CO_2_ in the water was in equilibrium with the CO_2_ in the headspace. The CO_2_ in the headspace gas was then separated by gas chromatography with helium carrier and analysed using a mass spectrometer. A range of DIC concentration solutions were prepared using sodium hydrogen carbonate for DIC concentration calibration. Three carbonate standards with known isotopic signatures (Across, Merck and Rennes II) were used for isotopic signature calibration^74^. The carbonate powders were loaded into 12 mL EXETAINER LABCO tubes with 0.1 mL distilled water before being flushed with helium and then analysed in the same way as the samples. DIC concentrations are expressed in mol L^−1^ with a reproducibility of ± 0.01 mol L^−1^, and isotopic signatures are expressed in ‰ relative to Vienna Pee Dee Belemnite (VPDB) with a reproducibility of ± 0.16 ‰ (1σ) (Supplementary Table S2).

#### Carbonate δ^13^C analysis

δ^13^C_carb_ was measured using a GasBench coupled to a Thermo Finnigan Delta^plus^ XP mass spectrometer (Thermo Fisher Scientific) for 57 sediment and stromatolite samples (Supplementary Table S3). Sediment samples were first frozen and lyophilized to remove water, then rinsed with ultrapure water and centrifuged three times to remove the salt and once again frozen and lyophilized. The rinsed and lyophilized sediments were ground with an agate mortar and pestle.

Several tests were carried out with samples only washed and ground, and with the same samples from which organic matter was removed by low-temperature oxygen-plasma ashing^75^ using a POLARON PT7160 RF system (Polaron Equipment Limited, Watford, UK). These tests showed that the organic matter in the untreated sample did not affect the isotope signature of the carbonates (Supplementary Table S3). Hence, the preparation and analysis of the carbonate samples was similar to that of the carbonate standards used for the isotopic calibration described above for DIC and δ^13^C_DIC_. The isotopic compositions are expressed in the delta notation, in ‰, relative to VPDB with a reproducibility of ± 0.2‰ (1σ) (Supplementary Table S3).

#### *Water POC and *δ^13^*C*_*POC*_* and sediment TOC and *δ^13^*C*_*org*_

The GF/F filters with the suspended particulate matter were decarbonated by exposure to concentrated HCl fumes in a desiccator for 12 h to avoid loss of material. The rinsed and lyophilized sediment samples were decarbonated with 1 N HCl in 50 mL Falcon tubes, then rinsed three times and centrifuged prior to analysis.

The POC and δ^13^C_POC_ on the decarbonated filters, and the TOC and δ^13^C_org_, in the decarbonated sediments were measured using a Flash EA1112 elemental analyser coupled to a Thermo Finnigan Delta^plus^ XP mass spectrometer via a Conflo IV interface (Thermo Fisher Scientific, Waltham, MA, USA) for 112 samples of particulate matter and sediment (Supplementary Tables S2 and S3). The decarbonated samples were loaded into tin capsules and heated to 1200 °C in a combustion tube with a mixture of chromium oxide and silver cobalt oxides. The combustion gases were carried by helium through a reduction column and a gas chromatography column to separate CO_2_ from the other gases. The CO_2_ was then injected into the mass spectrometer for isotopic analysis. For the isotope calibration, four internal standards of organic-rich soil or sediment were analysed in the same way. The concentrations are expressed as % with a reproducibility of 0.6% and the isotopic compositions as ‰ relative to VPDB with a reproducibility of ± 0.19‰ (1σ).

#### δ^13^C_CH4_ and δ^13^C_CO2_

δ^13^C_CH4_ and δ^13^C_CO2_ was measured on 28 samples (of lake water, sediment porewaters and gases bubbling up through the water column or diffusing at the water/air interface) using an Ultra Trace GC-Mass spectrometer (Supplementary Tables S2 to S4). Water samples were split between two EXETAINER LABCO tubes each containing 6 mL of water and 6 mL of helium. All samples were then agitated for three days to allow methane equilibration between the water and the headspace. Between 0.1 mL and 1 mL of gas was manually injected with a gas-tight syringe into the gas chromatograph to separate other gases from methane, which was then carried to the mass spectrometer for isotopic analysis. A CO_2_ and CH_4_ (1:1) internal standard with known isotopic compositions was injected for each analytical run three times before and three times after the samples. The isotopic compositions are expressed as ‰ relative to VPDB with a reproducibility of about ± 0.8‰ (1σ) (depending to the sample, details in the Supplementary Tables S2 to S4).

### Flux determination

#### Gross primary production from phytoplankton oxygenic photosynthesis and aerobic respiration

##### Photosynthesis parameters and respiration rates

Photosynthetic oxygen evolution was monitored as a proxy for photosynthetic activity. Samples collected from 0.25 to 1.5 m depth (April 2014) and from 0.5 m depth (October 2014) were distributed into air-tight 30 mL polycarbonate flasks placed in a custom-made controlled temperature photosynthetron with a light density gradient up to 1500 µmol photons m^−2^ s^−1^ using an array of 4 W white LEDs^76^. Photosynthetically active radiation (PAR) was measured using a US-SQS/L spherical quantum microprobe (Walz, Effeltrich, Germany)^76^. The temperature was kept the same as the lake by heating or cooling. Dissolved oxygen evolution corresponding to net primary production (photosynthetically evolved O_2_ minus respired O_2_) was measured every 20 min for 6 h of incubation using a FireSting optode system (Pyro-Science, Aachen, Germany)^76^. A set of triplicate samples was incubated in the dark at the same temperature, allowing respiration rate determination. Gross oxygen production rates were calculated for each of the light intensity applied, as the sum of the net oxygen production and absolute value of respiration rates. Carbon gross production and carbon respiration were expressed using a molar photosynthetic quotient C:O_2_ of 1 (ref.^77^) and plotted against irradiance.

##### Evaluation of lake rates of gross primary production

Production-irradiance (PI), incident photosynthetically active radiation (PAR), underwater light field, and diurnal carbon production were calculated using the Phytotools package for R (ref.^78^). Carbon gross production rates experimentally determined depending on light intensity were analysed using the model described by ref.^79^, and α (initial slope of PI relation at low PAR intensity), β (photoinhibition parameter at high PAR intensity) and Pmax (maximal production rate) parameters evaluated. Incident PAR was simulated, for the months of April and October, using the geographic coordinates of Dziani Dzaha, an atmospheric turbidity factor and shortwave radiation to PAR corrections^80^. Underwater light field and attenuation coefficient k were calculated from discrete field measurements of PAR performed at various depths during each survey using a Li-Cor LI-193 underwater spherical quantum sensor coupled to a LI-1400 data logger. Phytoplankton gross primary production (g C m^−2^ d^−1^) was calculated from simulated PAR at each depth for each month, with the measured attenuation coefficients and PI parameters as described above. The annual production rate was calculated by averaging these values over the whole year.

#### Organic carbon and carbonate burial fluxes

Organic carbon burial flux (*f*_burial_) was calculated based on TOC content and sediment accumulation rate, *ω*, which was determined as function of depth from the conservation of solid sediment mass as follows in Eq. (M1)^81^:

M1 $$\frac{\partial [\left(1-\phi \right)\omega ]}{\partial z}=0$$

where *z* is the depth (m) and *ϕ* is the porosity. The porosity decreases from 98% in the surface sediment to 80–85% at depth and is best described by a log distribution such as:

M2 $$\phi (z) = - 0.0199\ln (z) + 0.8188$$

Radiocarbon measurements of plant macro-remains in the sediment enabled to determine ages as function of depth^82^. The sedimentation rate follows a log distribution. It decreases drastically in the uppermost sediment and is almost constant below 20 cm of depth. A value of 1.2 mm year^−1^ is reached at one-meter depth. This value is used in our model to calculate the organic carbon and carbonate burial fluxes. Together with Eq. (M2), this burial rate was used to determine the sedimentation rate as function of depth by solving Eq. (M1).

Given a porosity between 98 and 90% for the top 40 cm and an organic carbon content between 10 and 20% in the rinsed and lyophilized sediment, depending on the core, the organic carbon burial flux is from 3.6 to 7.2 mmolC m^−2^ day^−1^ with an average value of 5.5 mmolC m^−2^ day^−1^ (i.e. 1.4% of gross primary production of 404 mmolC m^−2^ day^−1^). The carbonate carbon burial flux (*f*_carb_) was estimated from this organic carbon burial flux and the ratio of organic carbon to carbonate carbon in the sediments, with an average value of 1.9 mmolC m^−2^ day^−1^ (from 1.1 to 2.8 mmolC m^−2^ day^−1^).

#### CO_2_ and CH_4_ fluxes at the water/air interface

The CO_2_ flux (*f*_CO2_) and CH_4_ flux (*f*_CH4_) between the lake water and the atmosphere were estimated from several flux measurements at the water/air interface using a floating chamber^71,73^ with continuous CO_2_ and CH_4_ analysers (Licor LI-820 and Panterra Neodym respectively). In addition, air samples were taken from the floating chamber via a septum and stored in EXETAINER LABCO tubes for ulterior CH_4_ quantification by gas chromatography and Thermo-Conductor detector (Supplementary Table S5). The annual mean net CO_2_ degassing flux to the atmosphere at the water/air interface (*f*_deg_) was 123 mmolC m^−2^ day^−1^.

### Box model of the carbon cycle in Dziani Dzaha

We performed a basic box model of the carbon cycle in the lake (Fig. 2) to identify the effects of the flux (*f*) and carbon isotopic signature (δ^13^C) of each C input and output on the DIC carbon isotopic signature in the water column (δ^13^C_DIC_). The inputs include CO_2_ produced by methanogenesis (*f*_*CO2*-meth_ and δ^13^C_CO2-meth_), oxidation of methane produced by methanogenesis (*f*_*oxy*_ and δ^13^C_oxy_), aerobic and anaerobic respiration (*f*_*resp*_ and δ^13^C_resp_), detrital organic matter from watershed (*f*_det_ and δ^13^C_det_), and magmatic CO_2_ (*f*_mag_ and δ^13^C_mag_). The outputs include photosynthesis (*f*_ph_ and δ^13^C_ph_), carbonate burial (*f*_carb_ and δ^13^C_carb_) and net CO_2_ degassing into the atmosphere (*f*_deg_ and δ^13^C_deg_). Because of the stability of δ^13^C_DIC_ with time for a few decades (as recorded by the stability of the δ^13^C_carb_ and δ^13^C_org_ in the sediment cores), the system was assumed to be in a steady state and the box model can be expressed as a simple isotope mass balance equation (Eq. M3).

M3 $$f_{{{\text{input}}}} \times \delta^{{{13}}} {\text{C}}_{{{\text{input}}}} {-}f_{{{\text{output}}}} \times \delta^{{{13}}} {\text{C}}_{{{\text{output}}}} = \, 0$$

with *f*_input_ = *f*_output_.

This basic equation was developed by expanding the carbon inputs and outputs (Eq. M4).

M4 $$\begin{aligned} & f_{{{\text{resp}}}} \times\updelta ^{13} {\text{C}}_{{{\text{resp}}}} + f_{{{\text{CO2-meth}}}} \times\updelta ^{13} {\text{C}}_{{{\text{CO2-meth}}}} + f_{{{\text{oxy}}}} \times\updelta ^{13} {\text{C}}_{{{\text{oxy}}}} \\ & \quad \quad + f_{\det } \times\updelta ^{13} {\text{C}}_{\det } + f_{{{\text{mag}}}} \times\updelta ^{13} {\text{C}}_{{{\text{mag}}}} \\ & \quad = f_{{{\text{ph}}}} \times\updelta ^{13} {\text{C}}_{{{\text{ph}}}} + f_{\deg } \times\updelta ^{13} {\text{C}}_{\deg } + f_{{{\text{carb}}}} \times\updelta ^{13} {\text{C}}_{{{\text{carb}}}} \\ \end{aligned}$$

Equation M4 was developed to isolate the δ^13^C_DIC_ term, as previously proposed^56,58^ (Eqs. M5, M6):

M5 $$\begin{aligned} & f_{{{\text{resp}}}} \times (\updelta ^{13} {\text{C}}_{{{\text{DIC}}}} -\upvarepsilon _{{{\text{ph}}}} +\upvarepsilon _{{{\text{resp}}}} ) + f_{{\text{CO2 }{-}\text{ meth}}} \times (\updelta ^{13} {\text{C}}_{{{\text{DIC}}}} -\upvarepsilon _{{{\text{ph}}}} +\upvarepsilon _{{\text{CO2 }{-}\text{ meth}}} ) \\ & \quad \quad + f_{{{\text{oxy}}}} \times (\updelta ^{13} {\text{C}}_{{{\text{DIC}}}} -\upvarepsilon _{{{\text{ph}}}} { - \upvarepsilon }_{{\text{CH4 } - \text{ meth}}} -\upvarepsilon _{{{\text{oxy}}}} ) + f_{{\det}} \times\updelta ^{13} {\text{C}}_{{\det}} + f_{{{\text{mag}}}} \times\updelta ^{13} {\text{C}}_{{{\text{mag}}}} \\ & \quad = f_{{{\text{ph}}}} \times (\updelta ^{13} {\text{C}}_{{{\text{DIC}}}} -\upvarepsilon _{{{\text{ph}}}} ) + f_{\deg } \times (\updelta ^{13} {\text{C}}_{{{\text{DIC}}}} -\upvarepsilon _{\deg } ) + f_{{{\text{carb}}}} \times (\updelta ^{13} {\text{C}}_{{{\text{DIC}}}} -\upvarepsilon _{{{\text{carb}}}} ) \\ \end{aligned}$$

M6 $$\begin{aligned}\updelta ^{13} {\text{C}}_{{{\text{DIC}}}} & = f_{{{\text{resp}}}} \times\upvarepsilon _{{{\text{ph}}}} - f_{{{\text{resp}}}} \times\upvarepsilon _{{{\text{resp}}}} + f_{{\text{CO2-meth}}} \times\upvarepsilon _{{{\text{ph}}}} - f_{{\text{CO2-meth}}} \times\upvarepsilon _{{\text{CO2-meth}}} \\ & \quad + f_{{{\text{oxy}}}} \times\upvarepsilon _{{{\text{ph}}}} + f_{{{\text{oxy}}}} \times\upvarepsilon _{{\text{CH4 } - \text{ meth}}} + f_{{{\text{oxy}}}} \times\upvarepsilon _{{{\text{oxy}}}} - f_{\det } \times\updelta ^{13} {\text{C}}_{{\det}} - f_{{{\text{mag}}}} \times\updelta ^{13} {\text{C}}_{{{\text{mag}}}} \\ & \quad - f_{{{\text{ph}}}} \times\upvarepsilon _{{{\text{ph}}}} - f_{{\deg}} \times\upvarepsilon _{{\deg}} + f_{{{\text{carb}}}} \times\upvarepsilon _{{{\text{carb}}}} )/(f_{{{\text{resp}}}} + f_{{\text{CO2-meth}}} + f_{{{\text{oxy}}}} - f_{{{\text{ph}}}} - f_{\deg } - f_{{{\text{carb}}}} ) \\ \end{aligned}$$

In this final Eq. (M6), ε_*flux*_ is the isotopic enrichment factor for a given flux, derived either from a known isotope factor α (ε ≈ 1000 Ln α) or from the differences in isotope signatures between the source and sink pools. δ^13^C and ε are in ‰. All fluxes (*f*) are to and from the lake DIC pool and are expressed in mmolC m^−2^ day^−1^.

### Model constraints on fluxes and isotopic signatures

#### Fluxes determined by direct measurements

The fluxes of photosynthesis (*f*_ph_), aerobic respiration (*f*_resp_), CO_2_ and CH_4_ degassing into the atmosphere (*f*_deg_) and carbonate precipitation (*f*_carb_) were obtained by direct measurements (see details above).

#### Isotopic signature and enrichment factors determined by direct measurements

The isotopic signatures of the carbonates (δ^13^C_carb_), CO_2_ degassing into the atmosphere (δ^13^C_deg_), detrital organic matter (δ^13^C_det_), magmatic CO_2_ (δ^13^C_mag_), and particulate organic carbon (δ^13^C_POC_) were measured (see details above). These isotopic values were directly used to determine the enrichment factors (ε) associated with carbonate precipitation (ε_carb_), degassing of CO_2_ into the atmosphere (ε_deg_), detrital organic matter inputs (ε_det_), magmatic CO_2_ inputs (ε_mag_) and photosynthesis/respiration (ε_ph_ and ε_resp_).

#### Fluxes determined by isotopic mass balance calculation

The fluxes of CO_2_ generated by methanogenesis (*f*_CO2-meth_) and methane oxidation (*f*_oxy_) can be calculated using the mass balance equations expressed below (Eqs. M7,M8):

M7 $$f_{{{\text{meth}}}} = f_{{\text{CO2-meth}}} + f_{{\text{CH4 } - \text{ met}}}$$

M8 $$f_{{\text{CH4 } - \text{ meth}}} = f_{{{\text{oxy}}}} + f_{{\text{CH4 } - \text{ deg}}}$$

Taking into account a annual mean methane flux to the atmosphere of 77 mmolC m^−2^ d^−1^, and assuming that it represents 64% of the methane produced for a CO_2_:CH_4_ production ratio of 1:1, the CO_2_ flux produced by methanogenesis will be of about 120 mmolC m^−2^ d^−1^ and the one produced by CH_4_ oxidation will be of about 43 mmolC m^−2^ d^−1^.

Magmatic CO_2_ (*f*_mag_) and detrital organic matter remineralisation (*f*_det_): The contribution of DIC fluxes produced by detrital organic matter mineralisation and magmatic CO_2_ dissolution in the water column are the only unconstrained fluxes. The total of these fluxes can be calculated from the steady state mass balance equation (Eq. 1). The isotopic signature of the combined detrital organic matter and magmatic CO_2_ flux can also be calculated from the measured δ^13^C_DIC_ and equation (M6). Finally, using the average isotopic signatures of the detrital organic matter (− 26.7‰) and magmatic CO_2_ (− 2.7‰) the relative contribution of their respective fluxes was calculated from the isotopic mass balance equation below (Eq. M9, Supplementary Tables S4 and S6):

M9 $$f_{{\text{non-constrain}}} \times\updelta ^{13} {\text{C}}_{{\text{non-constrain}}} = f_{\det } \times\updelta ^{13} {\text{C}}_{{\det}} + f_{{{\text{mag}}}} \times\updelta ^{13} {\text{C}}_{{{\text{mag}}}}$$

This gives a detrital organic matter flux of 133 mmolC m^−2^ ^−^d^−1^ and a magmatic CO_2_ flux of 18 mmolC m^−2^ d^−1^. However, because these calculated fluxes depend strongly on the fraction of methane lost to the atmosphere, we tested the sensitivity of these values to changes in methane loss (see Supplementary material).

#### Isotopic signature and enrichment factors determined by isotopic mass balance calculation

##### CO_2_ generated by methanogenesis (δ^13^C_CO2-meth_/ε_CO2-meth_)

The sedimentary organic carbon isotopic signature was − 14.5 ± 0.7‰ and the methane dissolved carbon isotopic signature in pore water sediment was − 68.1 ± 2.3‰ (Supplementary Table S3). Using the isotopic mass balance equation below (Eq. M10) and assuming a CO_2_:CH_4_ production ratio of 1:1 for the organic matter degradation by methanogenesis pathway, the δ^13^C of methanogenic CO_2_ was calculated to 39.1‰. This isotopic signature was used to determine an enrichment factor associated with methanogenic CO_2_ production of 53.6‰.

M10 $$f_{{{\text{meth}}}} \times\updelta ^{13} {\text{C}}_{{{\text{SOC}}}} = f_{{\text{CO2-meth}}} \times\updelta ^{13} {\text{C}}_{{\text{CO2-meth}}} + f_{{\text{CH4 } - \text{ meth}}} \times\updelta ^{13} {\text{C}}_{{\text{CH4 } - \text{ meth}}}$$

However, because this value depends on the assumed CO_2_:CH_4_ production ratio of 1:1, the influence of this ratio was tested in Supplementary Information: CO_2_:CH_4_ production ratio.

##### CO_2_ generated by methane oxidation (δ^13^C_oxy_/ε_oxy_)

The methane carbon isotopic signature dissolved in the pore water sediment was − 68.1 ± 2.4‰ and the methane degassing into the atmosphere was − 65.4 ± 0.7‰ (Supplementary Tables S3 and S4), which indicate a slight isotope fractionation upon degassing. Using the isotopic mass balance equation below (Eq. M11) and assuming that 64% of the methane produced escapes into the atmosphere, the CO_2_ isotopic signature from methane oxidation was calculated to be of − 72.8‰. This isotopic signature was used to determine an enrichment factor associated with methane oxidation of 4.7‰.

M11 $$f_{{\text{CH4 } - \text{ meth}}} \times\updelta ^{13} {\text{C}}_{{\text{CH4 } - \text{ meth}}} = f_{{\text{CH4 } - \text{ deg}}} \times\updelta ^{13} {\text{C}}_{{\text{CH4 } - \text{ deg}}} + f_{{{\text{oxy}}}} \times\updelta ^{13} {\text{C}}_{{{\text{oxy}}}}$$

All fluxes and their isotopic signatures measured or constrained by isotopic mass balance equation are summarized in the Table 1. As all of the fluxes and isotopic signatures constrained by isotopic mass balance calculation strongly depend on the assumed proportion of methane escaping into the atmosphere and on the CO_2_:CH_4_ production ratio, the sensitivities of the parameters constrained by isotopic mass balance calculation to these assumption were tested with a CO_2_:CH_4_ production ratios of 1.2:1 and 1:1.2 and a methane loss to the atmosphere from 0 to 85% (Supplementary Information: CO_2_:CH_4_ production ratio and Methane loss). Overall, sensitivity tests were performed by sequentially changing each of the measured fluxes to evaluate their respective impact on δ^13^C_DIC_. To satisfy the steady state conditions, the detrital organic matter inputs and magmatic CO_2_ inputs were adjusted to maintain the mass balance, but maintaining their previously determined respective contributions (details in Supplementary Information).

## Supplementary information

Supplementary Information.

## Data Availability

All the data generated and analyzed in this study are available in the paper and in its Supplementary Information.
