# Supplementary material for: Carbon isotope evidence for large methane emissions to the Proterozoic atmosphere
Source: Sci Rep. 2020 Oct 23;10:18186. doi: 10.1038/s41598-020-75100-x (PMC7585424; doi:10.1038/s41598-020-75100-x)
Supplement: Supplementary file 1 — Supplementary Information. [file 41598_2020_75100_MOESM1_ESM.pdf]

Carbon isotope evidence for large methane emissions to the Proterozoic atmosphere

Pierre Cadeau[1*], Didier Jézéquel[1], Christophe Leboulanger[2], Eric Fouilland[2], Emilie Le Floc'h[2], Carine Chaduteau[1], Vincent Milesi[1], Julia Guélard[1], Gérard Sarazin[1], Amandine Katz[1], Sophie d'Amore[1], Cécile Bernard[3] and Magali Ader[1].

[1]Université de Paris, Institut de physique du globe de Paris, CNRS, F-75005 Paris, France

[2]MARBEC, Univ Montpellier, IRD, CNRS, Ifremer, 34200 Sète, France.

[3]Muséum National d'Histoire Naturelle, UMR MCAM, Paris, France.

[*]Correspondence to: pierre.cadeau@gmail.com

Supplementary Information

Additional context for the studied site

Sensitivity tests

Methanogenesis versus respiration

Photosynthesis

Carbon organic burial

CO_2 degassing into the atmosphere

Methane loss to the atmosphere

CO_2:CH_4 production ratio

Dependency between CH_4 loss, detrital and magmatic inputs

Supplementary Figures (Figure S1 to S5)

Supplementary Tables (Tables S1 to S6)

Additional context for the studied site

Dziani Dzaha ("crater lake" in the local language, Shimaore) is located in the Indian Ocean (12°46.237'S and 45°17.315'E) and probably results from one of the most recent eruptive events documented in the area, i.e. probably between 9000 and 4000 yBP)[29]. Its surface area is 2.36×10^5 m^2 and it is separated from the nearby seashore by a 230-meter thick crater wall. The lake is approximately at sea level and a bathymetric map was obtained during the April 2012 survey at the end of the wet season (Supplementary Figure S1). Except for a ~18m deep narrow depression, the maximum depth is ~4.5 to 5.2 meters, depending on the season.

Several surveys were conducted to investigate the lake's biological, physical and chemical characteristics (September 2010, September 2011, April 2012, April 2014, November 2014, April 2015, November 2015, and August 2016). Some of the results have been published and are summarized here[31-35].

The lake water is saline (up 70 psu, twice that of seawater), alkaline (alkalinity close to 0.2M, ~100 times that of seawater; the pH is between 9.0 and 9.8), and rich in dissolved inorganic carbon (up to 200 mM), with a relatively low sulphate concentration (0 to 3 mM) and a high dissolved organic carbon concentration (up to 9 mM). The daily averaged surface water temperature varies between 30°C and 36°C while that of the water at the lake bottom varies between 29°C and 31°C. This ecosystem is dominated by microorganisms, with a dense and perennial bloom of two photosynthetic species: the unicellular picoeukaryote *Picosystis salinarium* and the filamentous cyanobacteria *Arthrospira fusiformis*[34], the later accounting for more than 90% of the primary producer biomass and giving the lake a permanent green colour and high turbidity (particulate organic carbon concentration close to 7.5 mM)[31].

During the stratified period (December to July), meteoritic precipitation causes

stratification at around 2 meters deep. Below, neither dissolved oxygen nor sulphates are present and reduced chemical species accumulate (NH_4^+/NH_3 up to 0.5 mM, HS^-/H_2S up to 6 mM and CH_4 up to 2 mM). Active cyanobacteria are mainly found in the oxic surface waters and a dense and diverse population of archaea and heterotrophic bacteria dominates the biomass in these euxinic bottom waters[35].

During the non-stratified period (August to December) the lake is mixed and most physical, chemical and biological parameters are homogeneous with depth, except for dissolved oxygen which is only present down to about 1.5 m, depending on the photosynthetic activity during the daytime. Hardly any reduced chemical species accumulate below the oxycline and photosynthetic microorganisms are distributed throughout the water column.

Several bubbling sites have been identified within the lake, corresponding to magmatic degassing (Supplementary Figure S1). Stromatolites with various morphologies, mainly composed of calcite and aragonite, thrive in the shallow waters[33]. Away from the stromatolitic zones, the sediment is a varved organic–rich gel with carbonates[32].

Sensitivity tests

Methanogenesis versus respiration

Aerobic respiration is the only flux that could be directly measured (see Methods). It was of 214 mmolC m^{-2} day^{-1}. Because the anaerobic respiration flux was not determined in the course of this study, the aerobic respiration fluxes gives us the minimum value of the total respiration flux (f_{resp}). Its maximum value is given by the gross primary productivity (*i.e.* 404 mmolC m^{-2} day^{-1}) from which carbon burial is deduced (5.5 mmolC m^{-2} day^{-1}). In order to evaluate the minimum contribution of methanogenesis to the remineralisation of the primary production, we tested the sensitivity of the predicted values for $\delta^{13}C_{DIC}$ as a function of the relative proportions of mineralisation by methanogenesis and respiration[58].

Photosynthesis

The average oxygenic photosynthetic carbon fixation rate (f_{ph}) was of 404 mmolC m^{-2} day^{-1}. In the Supplementary Figure S2(A), the solid bold blue line shows $\delta^{13}C_{DIC}$ predicted using the measured and calculated parameter set including this value of oxygenic photosynthesis. The other lines are for oxygenic photosynthesis fluxes of 324, 364, 444 and 484 mmolC m^{-2} day^{-1}. An increase of 20% of the primary production to 484 mmolC m^{-2} day^{-1} corresponds to the upper limits of the flux measurement error. This sensitivity test suggests that an increase in photosynthetic flux alone is not sufficient to achieve the strongly positive $\delta^{13}C_{DIC}$ observed in the lake without methanogenesis. An increase of 20% of photosynthetic flux results in an increase of only 3.2‰ in $\delta^{13}C_{DIC}$ with methanogenesis but has no effect without it.

Carbon organic burial

The influence of organic carbon burial rate was tested for values between 0 mmolC m^{-2} day^{-1} and 28.3 mmolC m^{-2} day^{-1}. In the Supplementary Figure S2(B), the solid bold blue line shows $\delta^{13}C_{DIC}$ predicted using the measured and calculated parameter set with an organic carbon burial flux of 5.5 mmolC m^{-2} day^{-1}. The other lines are for organic carbon burial fluxes of. 0, 12.1, 20.2 and 28.3 mmolC m^{-2} day^{-1}. This sensitivity test suggests that an increase in organic carbon burial flux alone is not sufficient to achieve the strongly positive $\delta^{13}C_{DIC}$ observed in the lake. Without methanogenesis, an increase of 5.6% of carbon organic burial relative to gross primary production results in an increase of only 2.9‰ in $\delta^{13}C_{DIC}$.

CO_2 degassing into the atmosphere

The influence of this process was tested with values between 98.4 mmolC m^{-2} day^{-1} and 147.6 mmolC m^{-2} day^{-1}. These values correspond to an increase and decrease of about 20% of the mean measured fluxes. In the Supplementary Figure S2(C) the solid bold blue line shows $\delta^{13}C_{DIC}$ predicted using the measured and calculated parameter set including a CO_2 degassing flux of 123 mmolC m^{-2} day^{-1}. The other lines are for CO_2 degassing fluxes of 98.4, 110.7, 135.3 and 147.6 mmolC m^{-2} day^{-1}. The results of this sensitivity test are non intuitive and suggest that an increase of CO_2 degassing causes a decrease in $\delta^{13}C_{DIC}$. This is due to the steady state assumption implying that an increase of CO_2 degassing is compensated by an increase of detrital organic matter and magmatic CO_2 inputs. Because theses fluxes have negative isotopic signatures, the calculated $\delta^{13}C_{DIC}$ decreases. Without methanogenesis, no CO_2 degassing flux can explain the positive $\delta^{13}C_{DIC}$.

Methane loss

In the Supplementary Figure S3(A) the solid bold blue line shows $\delta^{13}C_{DIC}$ predicted using the measured and calculated parameters set considering a proportion of 64% of methanogenesis production lost to the atmosphere (see Table 1). The other lines are for methane losses of 0%, 45%, 75% and 85%. This sensitivity test suggests that an increase in the fraction of methane escaping into the atmosphere is an important parameter, the amount of strongly depleted CO_2 produced in the water column by oxidation of the remaining methane having a strong effect on $\delta^{13}C_{DIC}$. An increase of 21% in the proportion of methane lost to the atmosphere results in an increase of 4.6‰ in $\delta^{13}C_{DIC}$.

Methanogenesis CO_2:CH_4 production ratio

In the Supplementary Figure S3(B) the solid bold blue line shows $\delta^{13}C_{DIC}$ predicted using the measured and calculated parameter set assuming a CO_2:CH_4 methanogenesis production ratio of 1:1 (See Table 1). The other lines are for CO_2:CH_4 production ratios of 1.2:1 and 1:1.2. This sensitivity test suggests that a CO_2:CH_4 production ratio of 1:1.2 results in an increase of 1.6‰ of the $\delta^{13}C_{DIC}$.

Model dependency between CH_4 loss, detrital matter and magmatic inputs

We assumed that 64% of the methane produced by methanogenesis was lost to the atmosphere when calculating the CO_2 fluxes from methanogenesis and methane oxidation as well as when calculating the flux and isotopic signature of the combined detrital organic matter and magmatic CO_2 inputs. In previous sensitivity tests, we varied the fraction of methane lost to the atmosphere by adjusting detrital organic matter and magmatic CO_2 inputs to respect the mass balance flux without modifying their respective contribution. To test the influence of the fraction of methane lost to the atmosphere on the relative contributions of these two carbon sources and their impact on the predicted $\delta^{13}C_{DIC}$, we kept the initial CH_4 production by methanogenesis flux of 120 mmolC m^{-2} day^{-1} and varied the fraction of methane lost to the atmosphere, recalculating the isotopic signature of the combined detrital organic matter and magmatic CO_2 inputs to match the measured value of $\delta^{13}C_{DIC}$. Their relative contributions can be calculated from their combined isotopic signatures. The results are shown in the Supplementary Figure S4(A), with a maximum combined isotopic signature of -2.7‰ (100% magmatic CO_2) and minimum of -26.7‰ (100% detrital organic matter). The initial assumption 64% of methane produced by methanogenesis lost to the atmosphere gives an 88% contribution of detrital organic matter to the combined input ($\delta^{13}C$ = -23.5‰).

To test the influence of methane loss combined with the determination of the relative contribution of detrital organic matter and magmatic CO_2 inputs, a fraction of 22%, 50% and 78% of methane loss was tested (Supplementary Figure S4(B)). These percentages correspond to ranges of detrital organic matter contribution of 0%, 68% and 100%, respectively.

Decreasing the methane loss to 50% resulted in a maximum increase of approximately 5.3‰ in $\delta^{13}C_{DIC}$, which suggests that this is not sufficient to achieve the strongly positive values observed in the lake without methanogenesis. A decrease of methane loss to 22% resulted in a maximum increase of approximately 20.6‰ in $\delta^{13}C_{DIC}$ and gave a 5‰ isotopic signature without methanogenesis. Nevertheless, a methane loss of only 22% is not sufficient to achieve the strongly positive values observed in the lake without methanogenesis while a 22% methane loss to the atmosphere is probably far too low given the lake's characteristics, and a 100% contribution of magmatic CO_2 input with no detrital organic matter inputs is very unlikely given the dense vegetation cover in the Dziani Dzaha watershed.

Overall, all the sensitivity tests performed demonstrate that methanogenesis with a high loss of methane to the atmosphere is required to reach the strongly positive $\delta^{13}C_{DIC}$ observed in the lake.

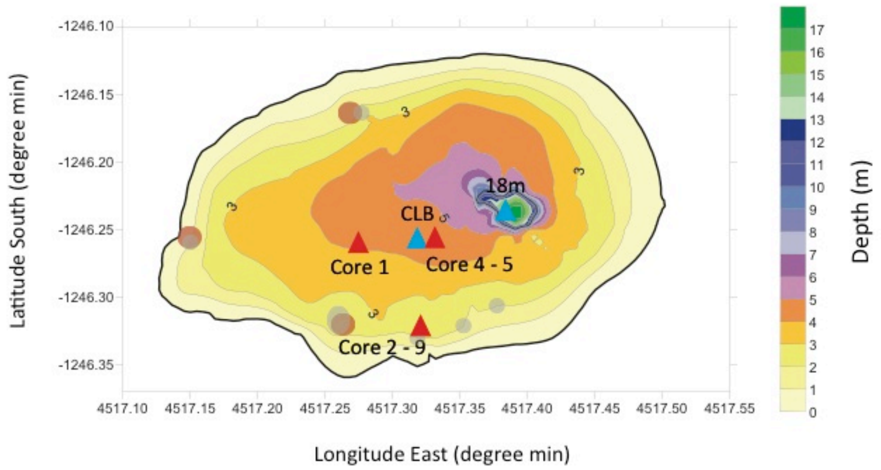

Supplementary Figure S1: Bathymetric map of the Dziani Dzaha lake, showing the different sampling areas. The red triangles represent the sediment core locations, the blue triangles represent the water column sampling locations, the red areas represent the identified bubbling areas at the water/air interface, and the grey areas represent occurrences of stromatolites (created by Didier Jezequel in 2012 from SURFER software, https://www.goldensoftware.com/products/surfer/trial).

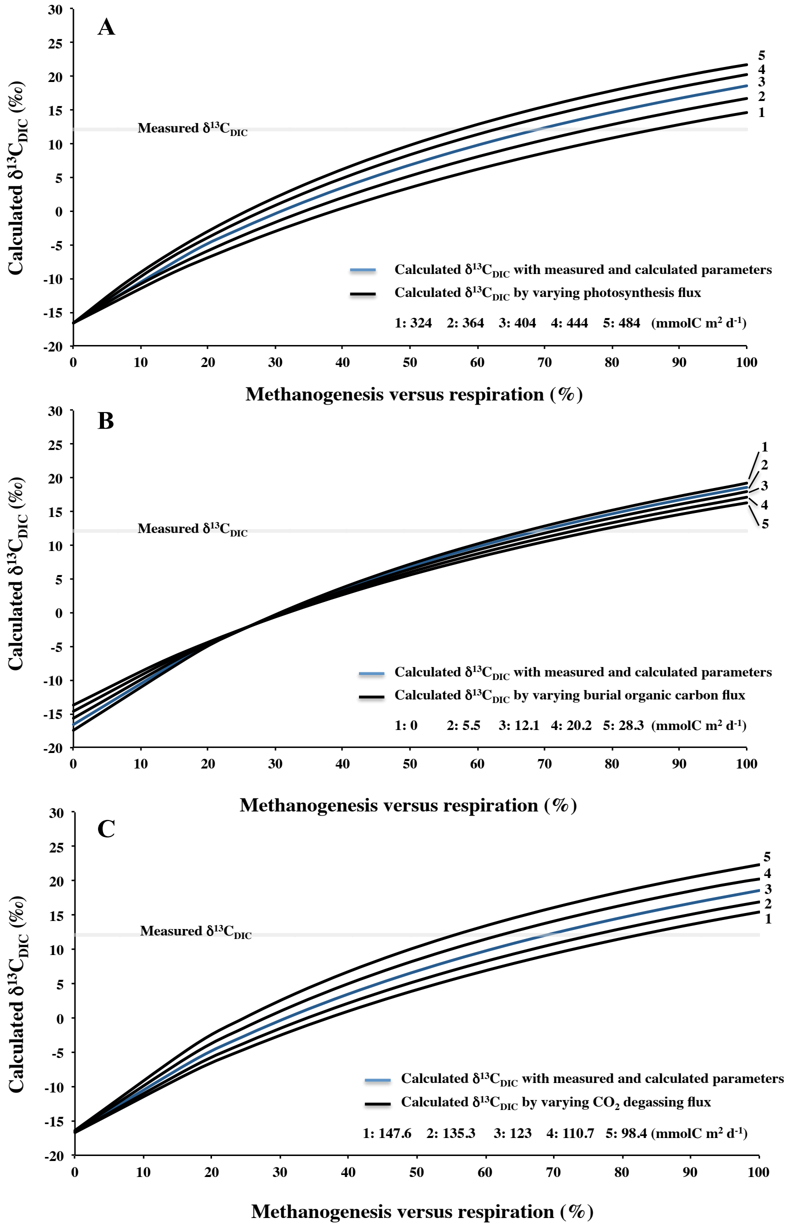

Supplementary Figure S2: Modelled $\delta^{13}C_{DIC}$ in the water column according to the proportion of organic carbon remineralized by respiration versus methanogenesis. From the bottom to the top, the different curves show: (A) different photosynthesis fluxes: 324 mmolC m^{-2} day^{-1}, 364 mmolC m^{-2} day^{-1}, 404 mmolC m^{-2} day^{-1} (blue curve), 444 mmolC m^{-2} day^{-1} and 484 mmolC m^{-2} day^{-1}, (B) different organic carbon burial fluxes: 0 mmolC m^{-2} day^{-1}, 5.5 mmolC m^{-2} day^{-1} (blue curve), 12.1 mmolC m^{-2} day^{-1}, 20.2 mmolC m^{-2} day^{-1} and 28.3 mmolC m^{-2} day^{-1} (at 0% of respiration *vs* methanogenesis), (C) different CO_2 degassing fluxes: 98.4 mmolC m^{-2} day^{-1}, 110.7 mmolC m^{-2} day^{-1}, 123 mmolC m^{-2} day^{-1} (blue curve), 135.3 mmolC m^{-2} day^{-1} and 147.6 mmolC m^{-2} day^{-1}. The grey band illustrates the averaged measured isotopic composition of DIC.

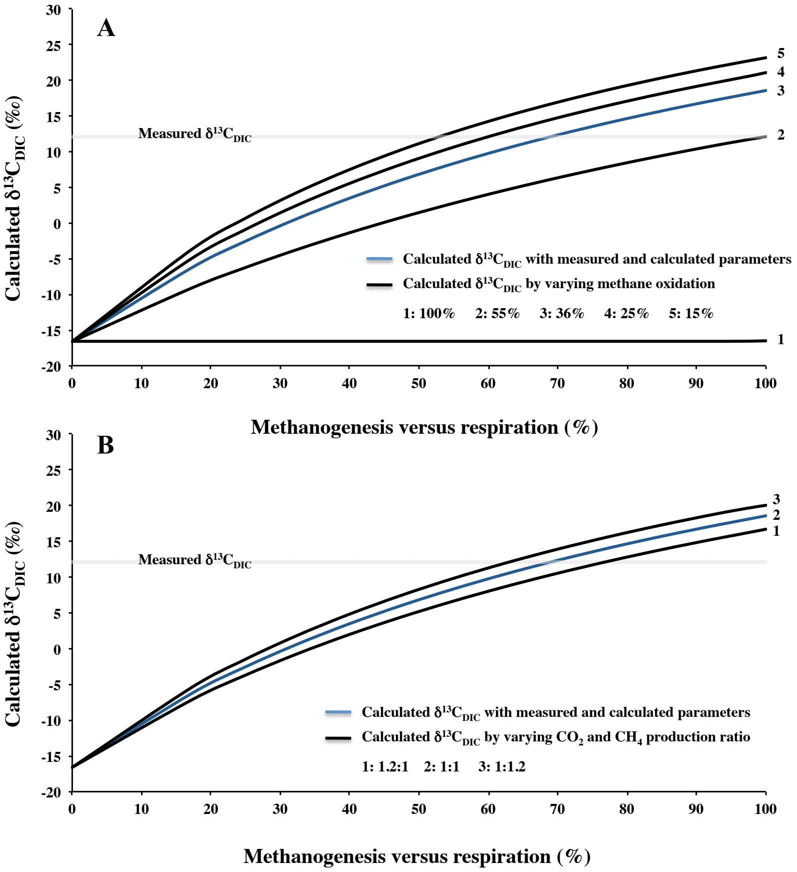

Supplementary Figure S3: Modelled $\delta^{13}C_{DIC}$ in the water column according to the proportion of organic carbon degraded by respiration versus methanogenesis. From the bottom to the top, the different curves show: (A) different percentage of methane oxidized in the water column: 100%, 55%, 36% (blue curve), 25% and 15%, (B) different CO_2 and CH_4 production ratio of about: 1.2:1, 1:1 (blue curve) and 1:1.2. The grey band illustrates the averaged measured isotopic composition of DIC.

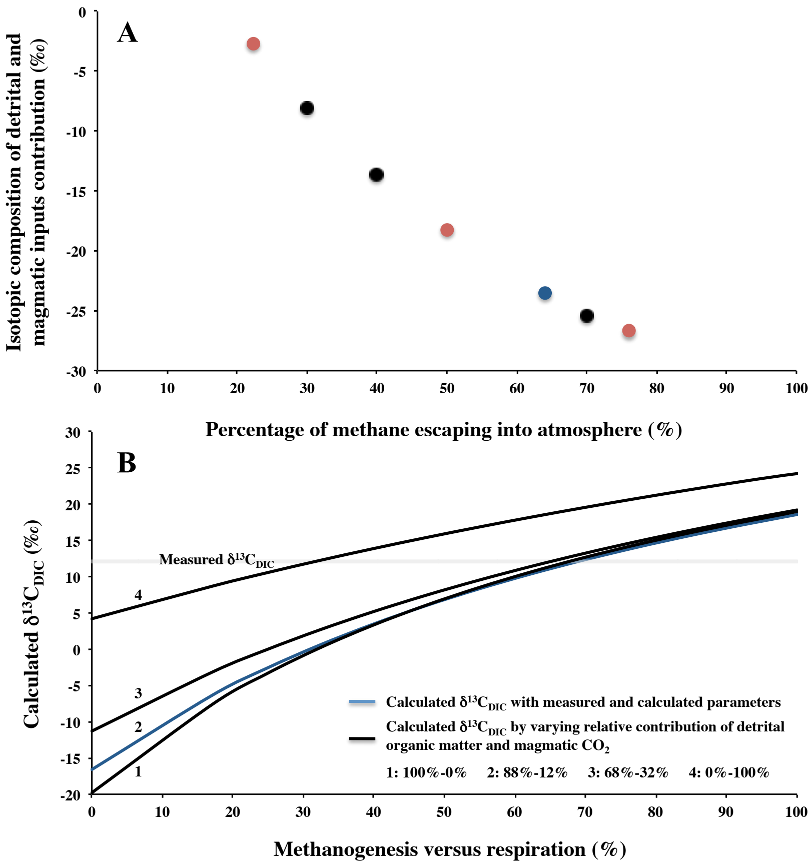

Supplementary Figure S4: (A) Modelled isotopic composition of the detrital and magmatic mixing required for completing the mass balance at steady state according to the percentage of methane loss to the atmosphere. The red dots represent the isotopic signature required for methane degassing to the atmosphere of 22%, 50% and 76%, with values of -2.7‰, -18.2‰ and -26.7‰ respectively (representing 100%, 32% and 0% of magmatic CO_2 contribution). (B) Modelled $\delta^{13}C_{DIC}$ in the water column according to the proportion of organic carbon remineralized by respiration versus methanogenesis. From the bottom to the top, the different curves show different relative contributions of detrital organic matter and magmatic CO_2 inputs: 100%-0%, 88%-12% (blue curve), 68%-32% and 0%-100%. The grey band illustrates the averaged measured isotopic composition of DIC.

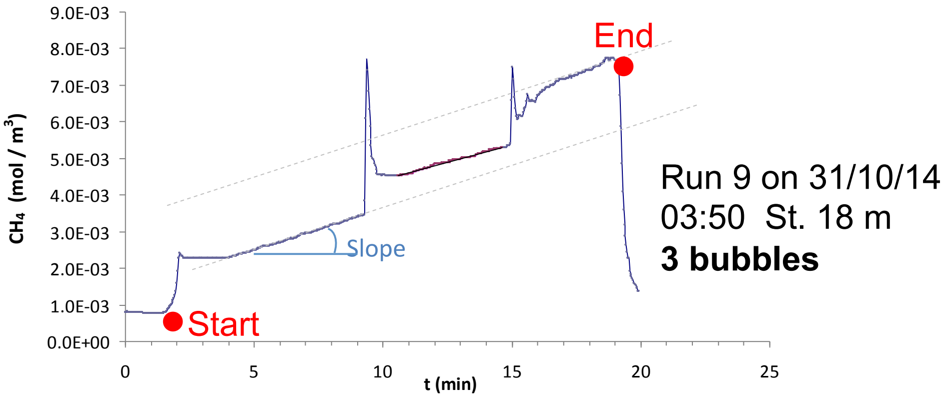

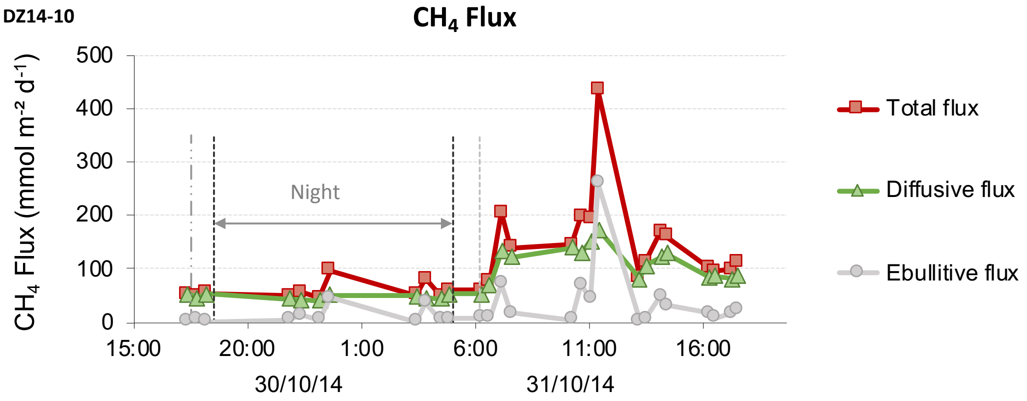

Ebullitive flux = 18 ± 15 % of total flux (n = 28; range 1–60%)

7

Supplementary Figure S5: Example of total, diffusive and ebullitive methane flux measurements using the floating chamber method (on the top). Total flux method "Start-End", with: Total methane flux =($CH_{4\ final}$ – $CH_{4\ initial}$) / Δ t; Diffusive flux = Slope × V/A; and Ebullitive flux = Total flux - Diffusive flux. V corresponds to the volume of the chamber and A corresponds to the area of air-water interface. Fluxes are in mmol m^{-2} d^{-1}. Example of 24h methane flux measurements, with total, diffusive and ebullitive fluxes (On the bottom).

Supplementary Table S1: Location of water column, sediment cores and stromatolites sampled in the Dziani Dzaha Lake.

	GPS coordinates (degree min)	
	Latitude South	Longitude East
Sediment cores		
DZ12-4 C1	S 12°46.269’	E 45°17.275’
DZ12-4 C2	S 12°46.325’	E 45°17.320’
DZ14-10 C4	S 12°46.260’	E 45°17.335’
DZ14-10 C5	S 12°46.260’	E 45°17.335’
DZ14-10 C9	S 12°46.325’	E 45°17.320’
Gas sample		
DZ15-10 1	S 12°46.259’	E 45°17.275’
DZ15-10 2	S 12°46.259’	E 45°17.275’
DZ14-10 G13	S 12°46.255’	E 45°17.150’
DZ14-10 G14	S 12°46.315’	E 45°17.255’
DZ14-10 pts3	S 12°46.170’	E 45°17.275’
Water column		
CLB	S 12°46.269’	E 45°17.315’
Stromatolites		
1-3	S 12°46.315’	E 45°17.255’
4-5	S 12°46.315’	E 45°17.350’
6	S 12°46.170’	E 45°17.273’

Supplementary Table S2: Concentration and carbon isotope composition of dissolved inorganic carbon, particulate organic carbon and dissolved methane in the water column of Dziani Dzaha Lake.

Depth (cm)	[DIC] (±0.01 mol/L)	$\delta^{13}C_{DIC}$ (±0.2‰)	$\delta^{13}C_{POC}$ (±0.2‰)	$\delta^{13}C_{CH4}$ (‰)	Deviation (‰)
DZ10-10 CLB					
0	0.17	12.4	-	-	-
0.25	0.17	12.4	-	-	-
0.5	0.17	12.4	-14.5	-	-
0.75	0.17	12.4	-14.2	-	-
1	0.17	12.4	-14.2	-	-
1.25	0.17	12.3	-14.1	-	-
1.5	0.18	12.4	-14.0	-	-
1.75	0.17	12.3	-14.2	-	-
2	0.17	12.3	-14.2	-	-
0.25	0.17	12.3	-14.3	-	-
2.5	0.17	12.3	-	-	-
2.75	0.17	12.3	-14.1	-	-
3	0.17	12.3	-14.4	-	-
3.25	0.17	12.3	-	-	-
3.5	0.17	12.3	-	-	-
3.75	0.17	12.3	-14.3	-	-
DZ11-10 CLB					
0	0.17	12.8	-	-	-
0.5	0.17	12.8	-14.5	-	-
1	0.17	12.7	-14.8	-	-
1.5	0.17	12.7	-14.2	-	-
2	0.17	12.7	-14.1	-	-
2.5	0.16	12.7	-14.0	-	-
3	0.17	12.5	-14.1	-	-
3.5	0.17	12.5	-	-	-
4	0.16	12.5	-14.1	-	-
DZ12-4 CLB					
0	0.13	13.3	-13.8	-	-
0.25	0.13	13.3	-14.3	-	-
0.5	0.13	13.3	-14.2	-	-
0.75	0.13	13.2	-14.0	-	-
1	0.13	13.1	-14.0	-	-

1.25	0.13	13.2	-14.0	-	-
1.5	0.12	13.1	-13.9	-	-
1.75	0.16	12.1	-13.4	-71.7	0.1
2	0.18	11.9	-13.3	-74.7	0.4
2.5	0.17	11.1	-12.8	-75.6	0.1
3	0.17	11.8	-12.7	-75.7	0.2
3.5	0.18	11.8	-12.9	-74.7	0.5
4	0.18	11.8	-	-72.9	0.2
4.5	0.17	11.8	-13.1	-72.1	0.1
DZ12-4 Station 18m					
0	-	-	-14.7	-	-
0.25	-	-	-14.7	-	-
0.5	-	-	-14.6	-	-
0.75	0.13	13.1	-14.7	-	-
1	0.13	13.0	-13.6	-	-
1.25	0.13	13.0	-14.6	-	-
1.5	0.20	11.8	-14.4	-	-
1.75	0.15	12.2	-14.2	-	-
2	0.13	13.0	-14.1	-	-
3	0.19	12.0	-12.3	-	-
5	0.13	13.2	-11.9	-	-
DZ14-4 CLB					
0.25	0.10	11.8	-14.0	-	-
0.75	0.10	11.6	-14.4	-	-
1	0.09	11.8	-14.5	-	-
1.25	0.10	11.7	-14.5	-	-
1.75	0.10	11.5	-13.8	-	-
2	0.16	10.9	-13.7	-	-
2.25	0.17	11.1	-12.0	-	-
2.75	0.20	11.1	-12.1	-	-
3.5	0.20	11.0	-12.3	-	-
DZ14-4 Station 18m					
0	0.10	12.0	-15.1	-70.9	0.8
0.25	0.10	11.9	-14.9	-	-
0.5	0.10	11.7	-14.8	-	-
0.75	0.10	11.6	-15.1	-	-
1	0.10	12.1	-14.9	-72.0	0.6
1.25	0.09	12.0	-14.8	-	-
1.5	0.00	12.0	-15.0	-72.9	0.4
1.75	0.11	11.7	-14.6	-	-

2	0.16	11.2	-14.4	-80.8	0.4
2.25	0.20	10.8	-14.4	-	-
2.5	0.19	11.2	-14.3	-	-
2.75	0.20	11.1	-17.4	-	-
3	0.18	11.1	-14.4	-	-
3.5	0.20	11.0	-13.6	-	-
4	0.20	10.9	-13.6	-	-
5	0.20	11.1	-13.7	-78.4	0.3
DZ14-10 CLB					
0.25	0.16	11.9	-15.0	-	-
0.5	0.17	12.2	-14.5	-62.6	0.3
0.75	0.16	12.4	-15.0	-	-
1	0.16	12.0	-15.1	-	-
1.25	0.17	12.1	-15.0	-	-
1.5	0.16	12.3	-15.0	-	-
2	0.16	12.5	-15.0	-	-
3	0.16	12.3	-15.0	-	-
3.5	0.16	12.0	-15.0	-63.0	0.6
DZ14-10 Station 18m					
0	0.18	12.3	-14.5	-70.0	0.2
1	0.18	12.2	-14.8	-	-
2.5	0.18	12.2	-14.5	-68.0	0.2
3.5	0.18	12.3	-14.0	-	-
5	0.19	12.2	-14.0	-66.6	0.1

Supplementary Table S3: Organic and inorganic carbon isotope composition of the sediment, interstitial pore-water and stromatolite sampled in the Dziani Dzaha Lake (see Supplementary table S1 for their coordinates).

Depth (cm)	$\delta^{13}C_{organic}$ (±0.2‰)	$\delta^{13}C_{carbonate}$ (±0.2‰)
DZ12-4 C1		
5 - 7.5	-15.6	-
7.5 - 8.5	-14.4	-
8.5 - 11	-14.2	-
12 - 16	-13.5	-
16 - 18	-14.9	-
18 - 22	-14.8	-
22 - 23	-15.0	-
23 - 28	-15.4	-
28 - 29	-16.0	-
29 - 34	-14.3	-
34 - 37	-13.6	-
37 - 40	-13.5	-
DZ12-4 C2		
2.5 - 4.5	-14.8	-
4.5 - 7	-14.8	-
7 - 10.5	-17.8	-
10.5 - 14	-18.5	-
14 - 16.5	-14.9	-
16.5 - 20	-14.1	-
20 - 23.5	-14.2	-
23.5 - 27	-14.3	-
27 - 30	-15.2	-
DZ14-10 C4		
0 - 1	-	14.3
11 - 14	-	17.8
24 - 27	-	14.7
30 - 35	-	16.9

Depth (cm)	$\delta^{13}C_{organic}$ (±0.2‰)	$\delta^{13}C_{carbonate}$ (±0.2‰)
DZ14-10 C9		
0 - 3.5	-	16.9
0 - 3.5	-	16.1
6 - 10	-	17.6
10 - 14	-	16.3
14 - 20	-14.1	-
20 - 24	-	16.1
32 - 36	-13.9	15.7
DZ14-10 C6		
0 - 3	-	16
0 – 3*	-	16.1
8.5-12	-	17.4
8.5-12*	-	17.2
16 - 20	-	15.8
16- 20*	-	15.9
36 - 40	-	16.9
36-40*	-	16.9
36 - 40	-	16.9
36-40*	-	16.8

Depth (cm)	$\delta^{13}C_{CH4}$ (‰)	Deviation (‰)
DZ14-4 C5		
3.5	-68.3	0.2
11	-67.0	0.2
18.5	-72.4	0.2
26	-65.9	0.2
33.5	-66.3	0.1
41	-68.8	0.4

Sample	$\delta^{13}C_{carbonate}$ (±0.2‰)
Stromatolite 1	
A	11.3
B	10.8
C	10.6
D	11.1
E	10.5
F	10.9
G	10.1
H	12.6
I	11.8
J	12.2
K	11.2
Stromatolite 2	
A	14.2
B	10.0
C	10.2
D	10.2
Stromatolite 3	
A	10.9
B	11.1
C	10.9
Stromatolite 4	
A	12.4
B	12.0
C	11.9
D	13.2
E	13.3
F	13.9

Sample	$\delta^{13}C_{carbonate}$ (±0.2‰)
Stromatolite 4	
G	13.8
H	11.3
I	10.6
J	12.1
K	11.7
L	11.2
M	11.1
Stromatolite 5	
A	10.4
B	10.9
C	10.9
D	11.1
E	10.7
F	10.4
G	10.6
H	10.7
Stromatolite 6	
A	13.5
B	11.2
C	10.0
D	12.3
E	11.4
F	11.9
G	10.6
H	10.8

* Carbon isotope composition of sedimentary carbonates after organic matter extraction by Low Temperature Ashing (LTA).

Supplementary Table S4: Carbon isotopic composition of CO_2 and CH_4 degassing into the atmosphere at the water/air interface in the Dziani Dzaha Lake.

Sample	$\delta^{13}C_{CO2}$ (±0.2‰)	$\delta^{13}C_{CH4}$ (±0.8‰)
DZ15-10 1	-	-65.2
DZ15-10 2	-	-65.7
DZ14-10 G13	-1.9	-
DZ14-10 G14	-2.4	-
DZ14-10 pts3	-3.8	-

Supplementary Table S5: CO_2 and CH_4 carbon fluxes at the water/air interface in the Dziani Dzaha Lake.

Date	CO_2 flux (mmolC m^2 day^{-1})			CH_4 flux (mmolC m^2 day^{-1})		
	Average	Minimum	Maximum	Average	Minimum	Maximum
-	-	-	-	-	-	-
April 2012	120	80	160	92	75	100
April 2014	42	33	49	136	60	300
April 2015	57	42	67	103	55	155
-	-	-	-	-	-	-
August 2016	60	49	76	107	56	176
-	-	-	-	-		-
October 2014	220	175	315	39	14	241
November 2015	211	184	243	38	14	88
November 2015	211	184	243	38	14	88
-	-	-	-	-	-	-
**Annual average**	**123**	**-**	**-**	**77**	**-**	**-**

Supplementary Table S6: Carbon isotope composition of higher plants collected around Dziani Dzaha Lake.

Sample (Name)	$\delta^{13}C_{organic}$ (‰)	Deviation (‰)
Ipomoea	-25.7	1.7
Palm tree	-25.5	1.7
Small tree	-26.4	1.5
Acacia	-28.0	1.5
Bulrush	-24.8	0.4
Banana tree	-28.0	0.9
Humus	-28.0	-
